# A Warning Against Antibiotic Treatment for Gastrointestinal Non-Typhoidal Salmonella Infections in Immunocompetent Adults: A Case Report and Review of the Literature

**DOI:** 10.7759/cureus.75953

**Published:** 2024-12-18

**Authors:** Elizabeth Keil

**Affiliations:** 1 Pharmacy, Providence Queen of the Valley Medical Center, Napa, USA

**Keywords:** antibiotics, antimicrobial stewardship, probiotics, public health, salmonella

## Abstract

*Salmonella enterica *is a common pathogen that causes foodborne illness worldwide. There is limited evidence describing the treatment of gastrointestinal non-typhoidal *Salmonella *(NTS). Clinicians are inclined to treat these infections with antibiotics, but the use of antibiotics may paradoxically worsen gastrointestinal symptoms and prolong bacterial stool shedding.

This is a case of gastrointestinal NTS in 30-year-old immunocompetent female patient. She experienced prolonged diarrhea for six weeks following food poisoning with *Salmonella enterica* Serovar Enteritidis and received antibiotics as a treatment for her symptoms. The antibiotics worsened her symptoms, and she continued to have positive stool tests. At week 11 following the initial infection, she was advised by her infectious diseases physician to avoid further antibiotic use and take probiotics including *Saccharomyces boulardii* and kefir probiotics. These improved her gastrointestinal symptoms; however, she continued to have bacterial stool shedding for a total of 25 weeks.

Antibiotics should be avoided for the treatment of gastrointestinal NTS in immunocompetent adults due to their lack of benefit and their potential for worsening and/or prolonging symptoms. Treatment should instead focus on recovering the gut microbiome.

## Introduction

*Salmonella enterica* is a gram-negative pathogen that is a worldwide public health threat. The Centers for Disease Control and Prevention estimate that *Salmonella* bacteria are responsible for 1.35 million infections, 26,500 hospitalizations and 420 deaths annually in the United States alone [1]. Certain patients who contract gastrointestinal *Salmonella* are required to follow up with public health. Depending on age and occupation, patients may have contact or work restrictions until they produce a negative stool test [2].

With newer technology for bacterial and viral identification via polymerase chain reaction (PCR), these pathogens can more easily be identified via a stool sample. Brendish et al. demonstrated quicker identification of gastrointestinal pathogens with PCR technology. However, this came at the cost of increased antibiotic use in the PCR group vs. the routine clinical care group (65% vs. 47%, p = 0.0028) [3].

There are certain patients who should be considered for antimicrobial treatment for gastrointestinal non-typhoidal *Salmonella* (NTS) including immunocompromised hosts, extremes of age (<3 months and >50 years of age), patients with certain conditions (valvular heart disease, atherosclerosis, malignancy, prosthetic material), and patients with extraintestinal disease [4]. While clinicians may reflexively prescribe antibiotics to treat all patients with gastrointestinal *Salmonella* infections, several studies have demonstrated a lack of benefit, an increased risk of persistent symptoms, and prolonged bacterial stool shedding in otherwise healthy patients who receive antibiotics [4-7]. This case demonstrates the harms associated with antibiotic treatment and the potential symptomatic benefits of probiotic treatment with kefir and *Saccharomyces boulardii* for gastrointestinal NTS infections.

## Case presentation

A 30-year-old female patient with no significant past medical history presented to the primary care clinic complaining of six weeks of diarrhea/loose stools, which originally started after food poisoning from sushi (Table 1). Upon the original onset of diarrhea, she had a low-grade fever for three days with a maximum temperature of 100 degrees Fahrenheit. The consistency of diarrhea was Bristol scale five or six, and she experienced two to nine episodes of diarrhea per day with fecal urgency. She denied watery stools. Five days prior to the food poisoning, she completed a seven-day course of cefixime (300 mg once daily) for a bacterial sinus infection. The patient had tried dietary changes including the banana, rice, applesauce, toast (BRAT) diet and over-the-counter bismuth subsalicylate with no relief.

**Table 1:** Timeline of Events BID, twice daily; BRAT, banana, rice, applesauce, toast; ID, infectious diseases; NTS, non-typhoidal *Salmonella; *PA, physician's assistant; PO, by mouth, SMX/TMP, sulfamethoxazole/trimethoprim

Week	Events	Treatment
-1	The patient finished a course of antibiotics for a bacterial sinus infection.	Cefixime 300 mg PO daily x seven days
1	Initial infection of gastrointestinal NTS from food poisoning.	BRAT Diet, Bismuth Subsalicylate PO as needed
2-5	The patient has ongoing diarrhea.	
6	The patient visits her primary care due to continued diarrhea and is diagnosed with gastrointestinal *Salmonella.* PA prescribes antibiotics. Stool sample is reported to public health.	SMX/TMP one DS tablet PO BID x seven days
7	The patient finishes antibiotics and diarrhea worsens (increased frequency, increased watery stool). The patient is referred to gastroenterology.	
8	Stool sample sent for culture and sensitivities. The patient tests negative for *Clostridioides difficile.* The patient sees the gastroenterologist who recommends starting a different antibiotic.	Levofloxacin 500 mg PO daily x seven days (declined by the patient)
9	The patient is referred to ID.	
11	The patient visits the ID specialist who recommends avoiding antibiotics and starting probiotic treatment.	*Saccharomyces boulardii* two capsules (500 mg) PO BID (2000 mg total/day), Lifeway® Kefir 1 cup PO daily
12	The patient’s diarrhea frequency and consistency improve.	*Saccharomyces boulardii *two capsules (500 mg) PO BID (2000 mg total/day), Lifeway® Kefir 1 cup PO daily
13-24	The patient has normal stools and consistency (two to three stools per day of solid stool). The patient’s stool continues to test positive for *Salmonella *during weekly tests with public health.	*Saccharomyces boulardii* two capsules (500 mg) PO BID (2000 mg total/day), Lifeway® Kefir 1 cup PO daily
25	The patient tests negative for *Salmonella.*	

The patient’s primary care physician’s assistant (PA) ordered a basic metabolic panel (BMP), complete blood count (CBC), the Verigene® Enteric Pathogen PCR (Luminex, Austin, TX, USA), and an Ova and Parasites smear. All tests returned as normal or negative except for the Enteric Pathogen PCR, which returned positive for *Salmonella* species. This result was reported to public health who further identified the organism as *Salmonella enterica* Serovar Enteritidis. Since the patient worked in healthcare, she was required to test negative prior to returning to work. The PA prescribed sulfamethoxazole/trimethoprim (SMX/TMP) 800/160 mg one double-strength tablet by mouth (PO) twice daily (BID) for seven days since the patient had long-term, ongoing symptoms after the initial food poisoning.

Following the course of antibiotics, the patient’s symptoms worsened. She reported an increased frequency of diarrhea and watery stools. Given worsening symptoms and recent antibiotic exposure, the patient was tested for *Clostridioides difficile* via the PCR/toxin reflex test, which returned negative. Susceptibilities for *Salmonella* were also requested due to concern for antibiotic resistance to SMX/TMP. However, the isolate was susceptible to all antibiotics tested: ampicillin, SMX/TMP and levofloxacin.

The patient was referred by her PA to a gastroenterologist who recommended restarting antibiotics with a different antibiotic based on susceptibility results (levofloxacin 500 mg PO daily x seven days). The patient declined further antibiotics given the worsening of her symptoms and asked for a referral to infectious diseases (ID). She remained symptomatic for the four weeks after completing her course of SMX/TMP with four to ten episodes of loose watery stools per day and continued to test positive for *Salmonella* during her weekly stool tests for public health.

The patient was further referred to ID who recommended avoiding further antibiotics. The ID provider also suggested starting an over-the-counter yeast probiotic, *Saccharomyces boulardii*, at a dose of two 500 mg capsules (1000 mg total) PO two times daily and a bacterial probiotic. The ID physician encouraged probiotic foods (such as kefir, sauerkraut, yogurt) with live cultures vs. over-the-counter options. The patient chose Lifeway® kefir and took one cup daily. Within the first week of starting *S. boulardii* and kefir the patient began to have solid stools and reported one to four solid stools per day with only intermittent loose stool over the next several weeks. The stool test remained positive for a total of 20 weeks following diagnosis (25 weeks total from the onset of diarrhea). She was cleared to return to work by public health.

## Discussion

Chronic typhoidal *Salmonella* carriage is well documented in the literature; however, persistent gastrointestinal NTS is less common. These infections are difficult to treat given their lack of response to antibiotics, and there is scant literature describing how to treat these infections [8]. Further, NTS can evade the host immune system by invading and replicating inside the host’s phagocytic and nonphagocytic cells [9]. Marzel et al. estimate that persistent gastrointestinal NTS occurs in around 2.2% of patients. Persistent infections were more common among younger patients (p=0.003), those who received antibiotics for their infection (p=0.018), those who were hospitalized (p=0.032), and those who were co-infected with other enteric pathogens (p<0.001). The persistence period ranged from 30 days to 8.3 years, and 65% of these patients had ongoing or relapsing diarrhea [7].

The microbiome plays a vital role in protecting against pathogenic enteric organisms such as *Salmonella*. Antibiotic treatment may damage or alter the host’s microbiome, which can increase the risk of infection and allow for *Salmonella* colonization and persistence. A study by Sekirov et al. demonstrated that mice treated with antibiotics (streptomycin and vancomycin) for two days prior to inoculation with *Salmonella enterica* resulted in an increased susceptibility to infection, higher rates of post-infection alterations in the microbiota, and more severe intestinal pathology [10]. The patient described in this case received a course of cefixime prior to her NTS infection, which may have initially disrupted her gut microbiome, and thus increased her susceptibility to *Salmonella*. The course of SMX/TMP during the infection also likely contributed to further microbiome depletion, increased *Salmonella* colonization, and persistence of symptoms.

*Saccharomyces boulardii* is a yeast probiotic which may have therapeutic benefit for infections caused by *Salmonella*. Several proposed mechanisms include protecting, tight junctions, binding pathogenic bacteria to yeast cells, inhibiting growth of bacteria, competitive exclusion, and preventing bacterial biofilm formation [11]. A small, randomized study by Krasnov in 73 patients with *Salmonella enterica* Serovar Enteritidis compared patients who received* S. boulardii* 500-750 mg orally daily with oral rehydration solution or oral rehydration solution alone. The mean frequency of stool was significantly lower in the patients who received *S. boulardii* vs. those who did not at 72-96 hours (1.4 vs 3.9, p = 0.02). They also noted a significant reduction in the time to first formed stool in the *S. boulardii* group vs. standard therapy (4.1 vs. 6.8 days, p = 0.02) and an increase in the levels of normal microflora bacterium from stool cultures collected from patients. Bacteria which had a statistically significant increase following the start of therapy included *Bifidobacterium, Lactobacillus*, and *Escherichia coli* (p = 0.05, 0.01, 0.02 respectively) [12].

Kefir is a fermented beverage formed from the kefir grain that includes a variety of bacterial and yeast probiotics. Traditional probiotics typically only contain one bacterial strain whereas kefir contains many, which can play a role in diversifying and regulating the microbiome. It is available in either milk or water beverages. Several microorganisms commonly found in kefir (including *Lactobacillus* species) have demonstrated antimicrobial activity against *Salmonella* and can inhibit its capacity to adhere to epithelial cells through competitive exclusion [11,13,14]. Since there are many kefir products available on the market with similar microorganisms, the patient in this case study was encouraged to choose whichever one she preferred.

While *S. boulardii* and other probiotics are generally well-tolerated and safe, there are potential risks in certain populations. Invasive infections due to *Lactobacillus* and *S. boulardii* have been described in patients taking probiotics who are immunocompromised, those with central lines, or those who may be susceptible to translocation of gut bacteria. Therefore, these may not be safe therapeutic options for these patients [15,16].

## Conclusions

This case reiterates the futility of antibiotic treatment for gastrointestinal infections secondary to NTS for otherwise healthy adult patients. This patient did not have any therapeutic benefit but experienced worsening symptoms following antibiotic therapy. Both her course of antibiotics prior to the *Salmonella* infection and for treatment of the infection may have contributed to persistent symptoms and prolonged stool shedding. Treatment of NTS should focus on recovering the gut microbiome since further dysbiosis may enable NTS to colonize the gut and persist. There is promising literature to support the use of *S. boulardii* and probiotics for reducing symptoms associated with NTS infections although it is unknown if they help shorten the time of bacterial stool shedding.
